# TRENDS AND FACTORS ASSOCIATED WITH PLACE OF DEATH AMONG INDIVIDUALS WITH CARDIOVASCULAR DISEASE IN THE UNITED STATES

**DOI:** 10.1093/geroni/igz038.482

**Published:** 2019-11-08

**Authors:** Sarah H Cross, Brystana G Kaufman, Haider Warraich

**Affiliations:** 1 Duke University, Durham, North Carolina, United States; 2 Duke-Margolis Center for Health Policy, Durham, North Carolina, United States; 3 Duke University, North Carolina, United States

## Abstract

While most patients prefer to die at home, trends and factors associated with place of death for patients dying of cardiovascular disease (CVD) remain unknown. Using data from the National Center for Health Statistics from 2003-2017, we described trends and conducted multivariable logistic regression to evaluate associations between demographic characteristics and place of death among CVD patients in the United States. From 2003-2017, the rate of CVD deaths occurring at home increased from 21.3% to 30.9%, and rate of hospice facility deaths increased from practically none to 6.0%. Over the same period, the rate of hospital deaths decreased from 36.5% to 27.3%, and nursing facility deaths decreased from 25.1% to 20.6%. With the exception of conduction disorders, temporal trends in place of death were consistent across CVD diagnosis subgroups: ischemic heart disease, hypertensive heart disease, heart failure/cardiomyopathy, cerebrovascular disease, aortic stenosis, and all other CVDs. Differences between demographic groups persisted over the study period, with reduced odds of home death among Hispanic versus non-Hispanic (OR=.942; 95% CI .929-.955) decedents, Black versus White (OR=.837; CI .809-.866) decedents and greater odds of home death among decedents with some college education or more (OR=1.08; CI 1.06-1.09) versus decedents with a high-school education or less. In 2014, home surpassed hospital as the most common place of death for CVD patients. CVD patients often have acute and intense needs at the end of life that are challenging to manage in the home and the quality of care these patients receive should be further investigated.

